# Supplementary material for: Identification of drug-specific public TCR driving severe cutaneous adverse reactions
Source: Nat Commun. 2019 Aug 8;10:3569. doi: 10.1038/s41467-019-11396-2 (PMC6687717; doi:10.1038/s41467-019-11396-2)
Supplement: Supplementary file 1 — Supplementary Information [file 41467_2019_11396_MOESM1_ESM.pdf]

Supplementary Information for

Identification of drug-specific public TCR driving severe cutaneous adverse reactions

Pan *et al*.

This PDF file includes:

Supplementary Fig. 1-13

Supplementary Table 1-8

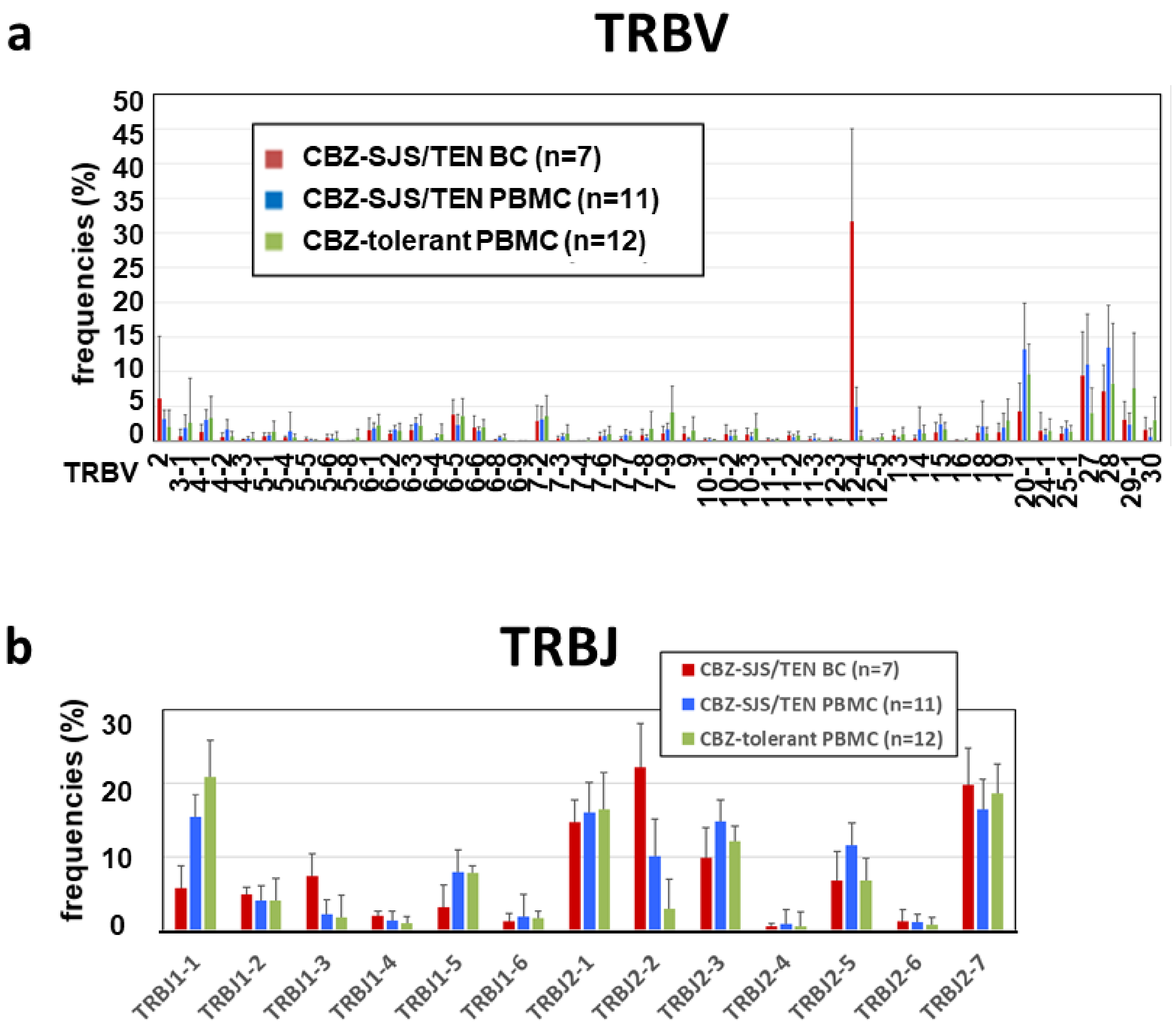

Supplementary Figure 1. T cell receptor β variable (*TRBV*) and T cell receptor β joining (*TRBJ*) gene usage in the blister cells or PBMC of CBZ-SJS/TEN patients and tolerant controls. The bars represent the mean frequencies of respective *TRBV* (**a**) and *TRBJ* (***b***) gene usage in the blister cells (n=7, red) and PBMC (n=11, blue) from patients with CBZ-SJS/TEN, and the PBMC from CBZ tolerant-controls (n=12, green). The results are expressed as mean ±s.e.m.

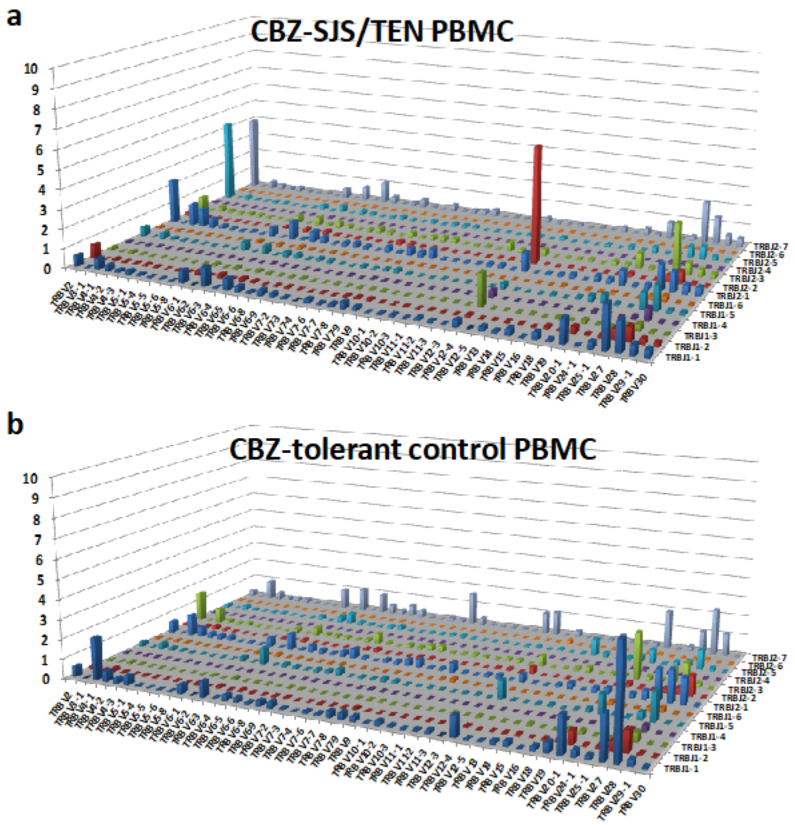

Supplementary Figure 2.*TRBV*/*TRBJ* gene pairing in PBMC of patients with CBZ-SJS/TEN, and CBZ-tolerant controls. The *TRBV*/*TRBJ* gene pairing of PBMC of 11 patients with CBZ-SJS/TEN (**a**), and 12 CBZ-tolerant controls (**b**) is shown in the 3-dimensional histograms. The *x*- and *y*-axis represent the TRBV and TRBJ regions, respectively, and the *z*-axis indicates the mean frequencies of TCRβ rearrangements detected by high-throughput NGS.

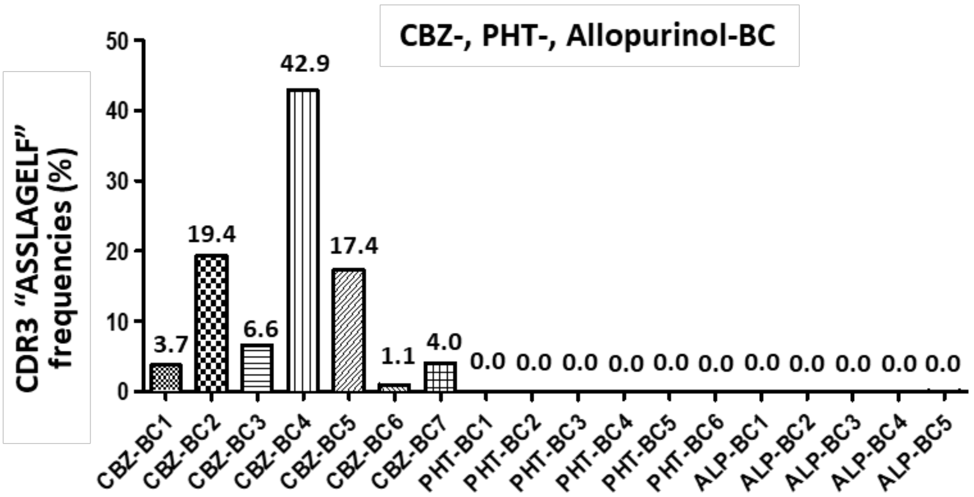

Supplementary Figure 3. The frequencies of TCRβ CDR3 clonotype "ASSLAGELF" in the blister cells of patients with SJS/TEN. The *y*-axis shows the frequencies of TCRβ CDR3 "ASSLAGELF" in the samples detected by high-throughput NGS. The *x*-axis shows the data of blister cells (BC) from patients with SJS/TEN caused by CBZ (carbamazepine), PHT (phenytoin), and ALP (allopurinol).

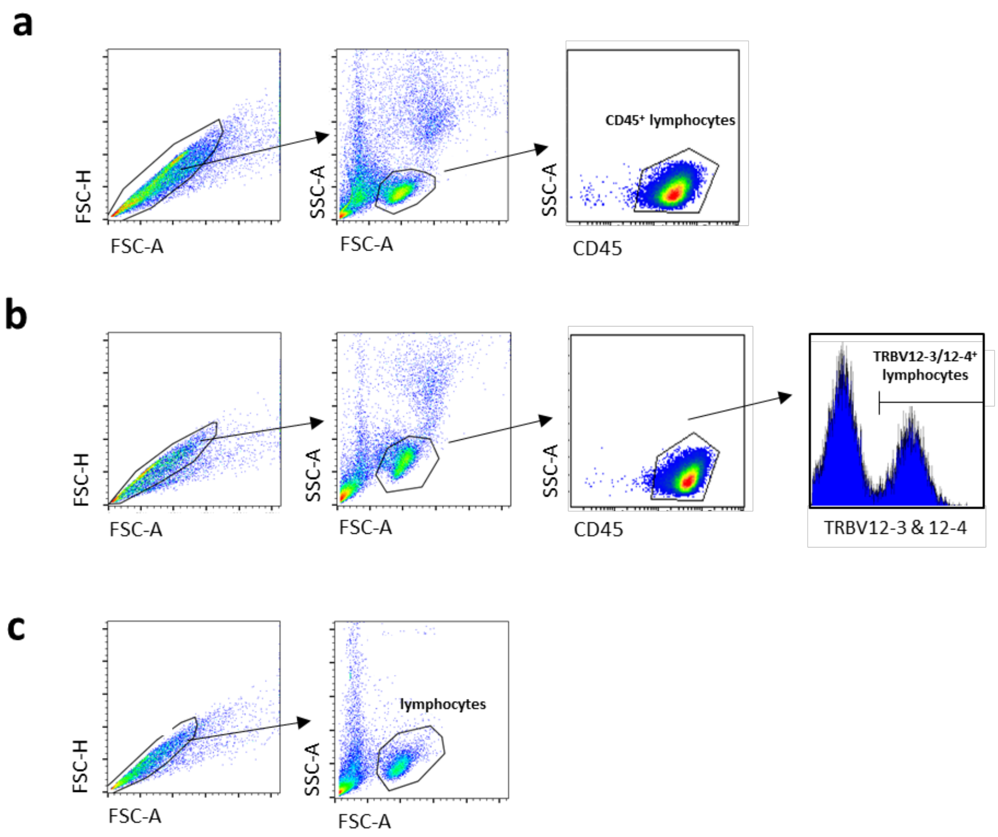

Supplementary Figure 4. Gating strategies used for flow cytometry. (a) Gating strategy for CD45+ lymphocytes from blister cells of patients with CBZ-SJS/TEN for cell populations analysis presented on Fig. 2g. (b) Gating strategy for TRBV12-3/12-4+ lymphocytes (CD45+) from blister cells of patients with CBZ-SJS/TEN for cell populations analysis and flow sorting presented on Fig. 2h, 4, and Supplementary Figure 6. (c) Gating strategy for lymphocytes from mouse PBMC for cell population analysis presented on Fig. 7f-j.

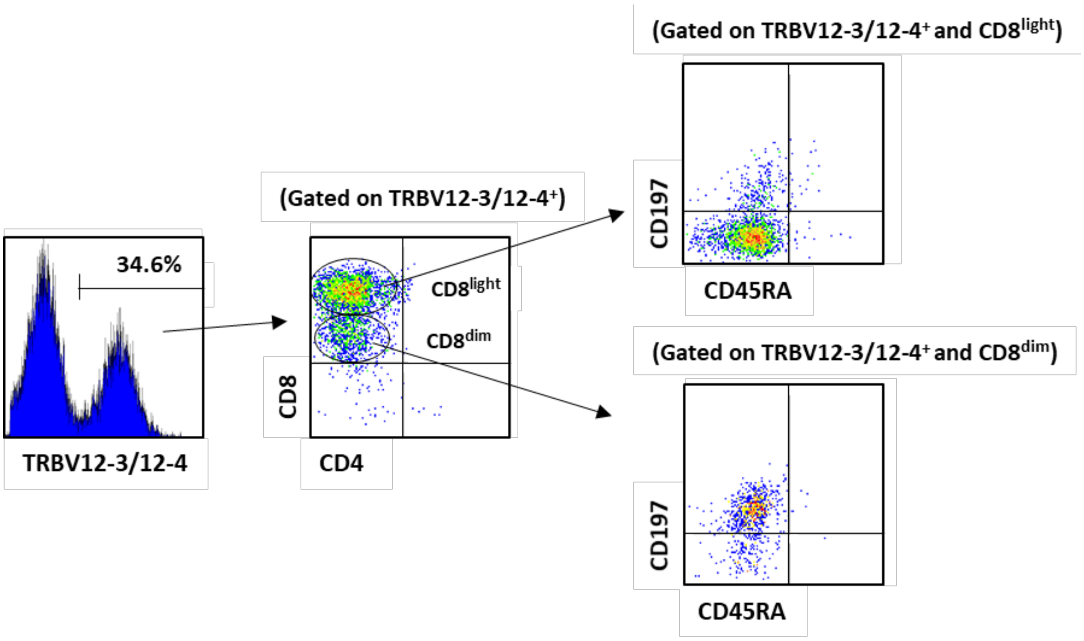

Supplementary Figure 5. Analysis of the cell markers of TRBV12-4(+) blister cells. The representative flow cytometry data show that 34.6% of the blister cells were stained by antibody against TRBV12-3/12-4 in a patient with CBZ-SJS/TEN (case 4). When gated on these cells expressing TRBV12-3/12-4, the $CD8^{light}$ cells (71.7%) were mainly effector memory T cells ($CD197^{-}CD45RA^{-}$) and the $CD8^{dim}$ cells (22.9%) were mainly central memory T cells ($CD197^{+}CD45RA^{-}$).

a

CBZ-BC4_TCRβ:

	CDR3 clonotype	TRBV	TRBJ	Frequency (%)
1	ASSLAGELF	V12-4	J2-2	42.85
2	ASSYRDGYEQY	V6-5	J2-7	2.73
3	ASSRRAVVGSYNEQF	V7-2	J2-1	2.62
4	ATHGTGYLEQY	V25-1	J2-7	2.15
5	ASSLSGELF	V12-4	J2-2	1.77

CBZ-BC4_TCRα:

	CDR3 clonotype	TRAV	TRAJ	Frequency (%)
1	VFDNTDKLI	V12-1	J34	44.81
2	AASPPDGNQFY	V23/DV6	J49	6.58
3	AATPPDGNQFY	V23/DV6	J49	2.05
4	AVNADSGATNKLI	V8-1	J32	1.44
5	VVEKSAGNMLT	V12-1	J39	1.04

b

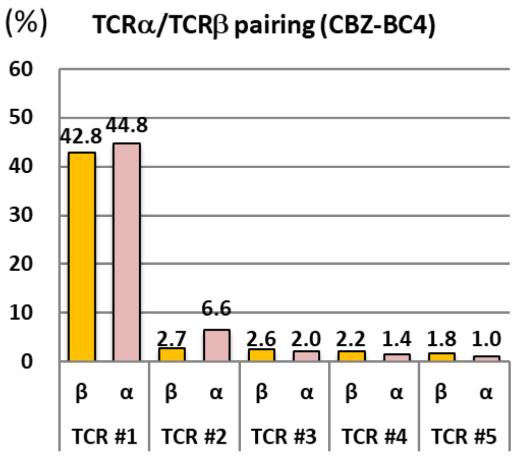

Supplementary Figure 6. The TCRα and TCRβ repertoire of the blister cells of a representative patient with CBZ-SJS/TEN. The TCRα and TCRβ repertoire of blister cells of a representative patient with CBZ-SJS/TEN (case 4) were analyzed by high-throughput next-generation sequencing. (**a**) The five most common TCRβ CDR3 clonotypes (shown in orange) and TCRα CDR3 clonotypes (shown in pink), and their corresponding variable/joining types and respective frequencies are listed. (**b**) The paired TCRa and TCRb clonotypes were suggested by the order of frequency ranking, and the most common TCRα CDR3 clonotype "VFDNTDKLI" and TCRβ CDR3 clonotype "ASSLAGELF" accounted for 44.81% and 42.85% of total reads of TCRα and TCRβ CDR3 clonotypes, respectively, were paired.

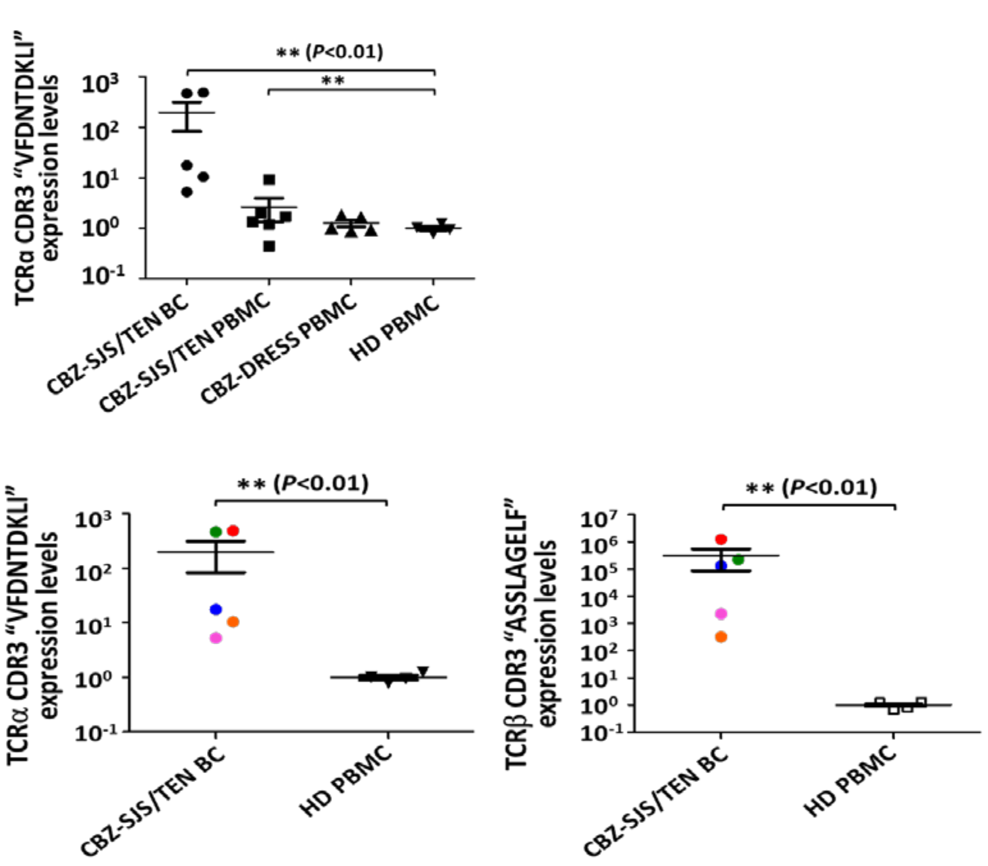

Supplementary Figure 7. Expression of the specific TCRα CDR3 clonotype "VFDNTDKLI" in the blister cells or PBMC samples of CBZ-SJS/TEN patients, CBZ-DRESS patients, and healthy donor (HD) controls. (**a**) The expression of TCRα CDR3 clonotype "VFDNTDKLI" was determined by TaqMan quantitative real-time PCR using the samples of the blister cells of CBZ-SJS/TEN patients (n=5), or PBMC of CBZ-SJS/TEN patients (n=6), CBZ-DRESS patients (n=5) and health donors (HD) (n=4). The TaqMan real-time PCR assay for detecting the dominant TCRα clonotype "VFDNTDKLI" was designed as described in ***Materials and Methods***. The expression level of the specific TCRα clonotype was normalized by that of CD3, and the detection limit of the TCRα clonotype/CD3 ratio was 0.0001. (**b**) The expression levels of TCRα CDR3 "VFDNTDKLI" and TCRβ CDR3 "ASSLAGELF" were compared in different samples of blister cells from CBZ-SJS/TEN patients and PBMC of healthy donors (HD). Similar expression trend of CDR3 "VFDNTDKLI" and TCRβ CDR3 "ASSLAGELF" was found in different blister cell samples. The results are expressed as mean ± s.e.m. with each dot representing the data of an individual. Statistical analysis was generated using an unpaired, two-tailed Student's *t* test. **P < 0.01.

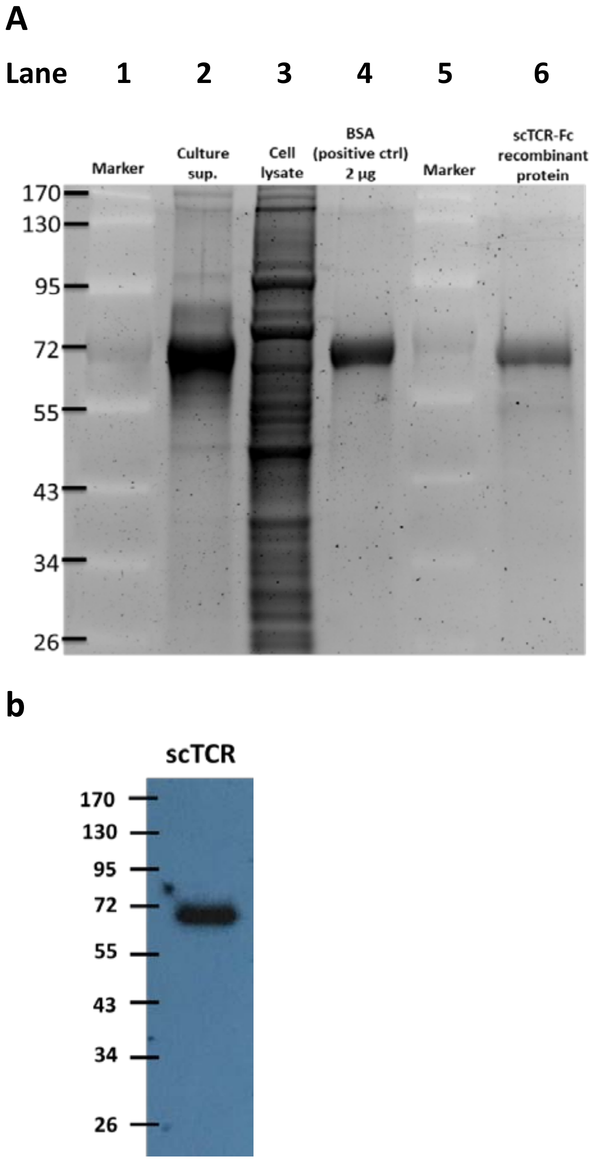

Supplementary Figure 8. SDS-PAGE, SYPRO Ruby staining and Western blot analyses of the purified single-chain αβTCR (scTCR) recombinant protein. (a) The SDS-PAGE and SYPRO Ruby staining show that the scTCR-Fc recombinant protein with a molecular weight of approximately 70kDa accounted for the major protein in the serum-free cultured medium of the culture supernatant of HEK293F cells transfected with scTCR-Fc plasmid (lane 2). The scTCR-Fc recombinant protein was purified by protein A beads, and analyzed by the SDS-PAGE and SYPRO Ruby staining (lane 6). The proportion of scTCR-Fc in the culture medium (lane 2) and purified recombinant protein (lane 6) was estimated by image J software, and showed 92.4% and 98% of the total proteins of the samples, respectively. (**b**) Detection of the scTCR-Fc recombinant protein by Western blot using anti-human IgG (Fc) antibody (Millipore).

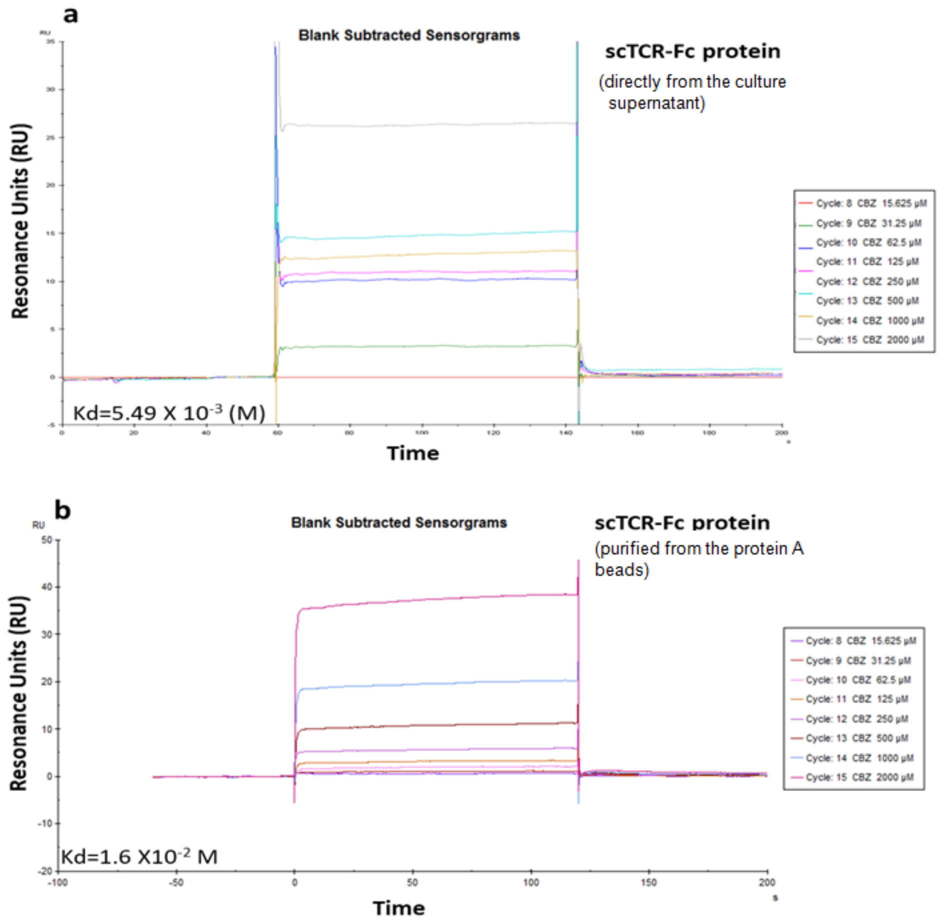

Supplementary Figure 9. The SPR binding response of the public αβTCR recombinant protein to CBZ. As the proportions of scTCR-Fc in the culture medium or the purified recombinant protein eluted from protein A beads were very high, we analyzed the binding response and activity of the protein samples from both sources by Biacore T200 SPR assay. (**a**). The binding response was observed using the protein sample directly from the culture supernatant of the public scTCR-Fc plasmid-transfected HEK293F cells. (**b**). The binding reponse of the scTCR protein purified by the protein A beads. The drug flew through the chip, and the binding response was measured. The binding response of the protein from either source was similar. The measured Kd values of scTCR-Fc protein from culture supernatant and eluted from protein A beads were 5.49x10^{-3} M and 1.6 x10^{-2} M, respectively.

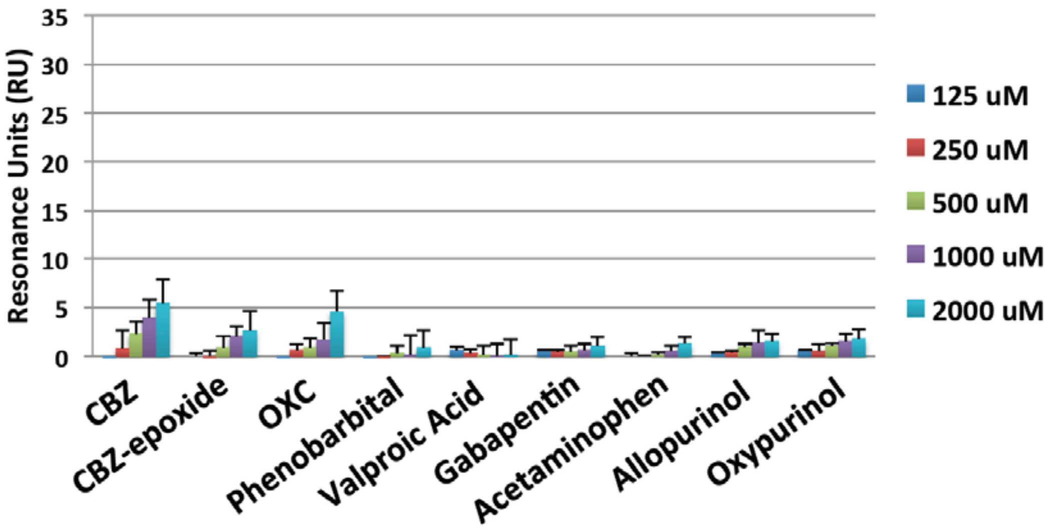

Supplementary Figure 10. The control αβTCR recombinant protein shows no binding response to CBZ or other drugs. The TCRα and TCRβ repertoire of the blister cells of a CBZ-SJS/TEN patient (case 6) was analyzed by high-throughput next-generation sequencing. The TCRα CDR3 "AGHDYKLS" and TCRβ CDR3 "ASTSGPNEQF" clonotypes accounted for 10.52% and 7.74% of all TCRα and TCRβ CDR3 reads, respectively, were selected. The cDNA encoding these two TCRα and TCRβ clonotypes was cloned into the control scTCR construct, and transfected to HEK293F cells. The control scTCR recombinant protein was purified and coated on the chip of the Biacore T200 surface plasmon resonance (SPR) system. The binding response of the control scTCR recombinant protein to CBZ and different drugs was examined. The results are representative of three independent experiments, and expressed as mean ± s.e.m.

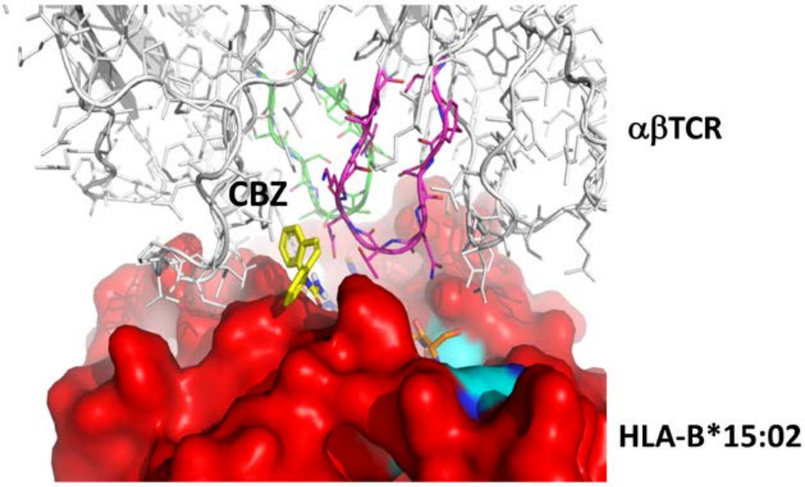

Supplementary Figure 11. *In silico* molecular modeling of the interaction of the public αβTCR, carbamazepine, and HLA-B*15:02 protein. Molecular docking suggested that CBZ (yellow for carbon) was more likely to bind the solvent exposed portion of the interface between the public αβTCR and HLA-B*15:02, which was comprised of the α1 helix of HLA-B*15:02 (red), TCRα CDR3 (magenta), and TCR β CDR2 (grey).

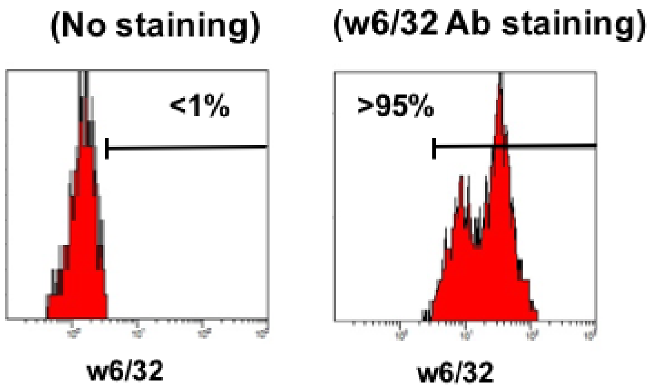

Supplementary Figure 12. Stable expression of the HLA-B*15:02 molecule in the transgenic mice. The peripheral blood mononuclear cells (PBMC) of *HLA-B*15:02* transgenic mice were isolated and stained by anti-human HLA class 1 Ab (clone: w6/32) to confirm the expression of human HLA. The flow cytometry results showed that more than 95% of cells from the PBMC of *HLA-B*15:02* transgenic mice stably expressed the HLA molecule.

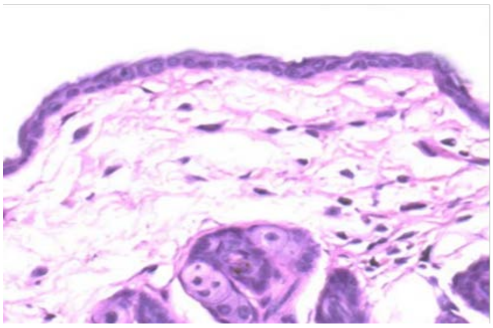

Supplementary Figure 13. Histology of skin biopsies of the HLA-B*15:02$^+$TCR$^+$CBZ$^+$ mice (group III). The HLA-B*15:02 transgenic mice received oral administration of carbamazepine and adoptive transfer of the public αβTCR-transfected T lymphocytes as described in ***Materials and Methods***. The H&E staining of the affected skin biopsies showed marked dermal inflammatory cell infiltration with epidermal dyskeratosis.

Supplementary Table 1. Demographics and *HLA-A* and *HLA-B* genotypes of enrolled patients with CBZ-induced SJS/TEN

Patient ID	Culprit drug	Clinical diagnosis	Samples	Disease Stage for experiments	*HLA-A* genotype	*HLA-B* genotype	Populations
Case1	CBZ	SJS/TEN	BC	active	A*11:01 / A*11:01	B*15:02 / B*13:01	Han Chinese
Case2	CBZ	SJS	BC, PBMC	active	A*02:03 / A*11:01	B*15:02 / B*13:02	Han Chinese
Case3	CBZ	SJS	BC, PBMC	active	A*11:01 / A*33:03	B*15:02 / B*54:01	Han Chinese
Case4	CBZ	TEN	BC, PBMC	active	A*11:01 / A*24:02	B*15:02 / B*51:01	Han Chinese
Case5	CBZ	SJS/TEN	BC, PBMC	active	A*11:01 / A*11:01	B*15:02 / B*15:02	Han Chinese
Case6	CBZ	SJS/TEN	BC, PBMC	active	A*11:02 / A*32:01	B*15:02 / B*38:02	Han Chinese
Case7	CBZ	TEN	BC, PBMC	active	A*11:01 / A*11:01	B*15:02 / B*55:02	Han Chinese
Case8	CBZ	SJS	PBMC	active	A*11:01 / A*11:01	B*15:02 / B*13:01	Han Chinese
Case9	CBZ	SJS	PBMC	active	A*02:03 / A*24:02	B*15:02 / B*40:01	Han Chinese
Case10	CBZ	TEN	PBMC	recovery	A*11:01 / A*11:01	B*15:02 / B*58:01	Han Chinese
Case11	CBZ	SJS	PBMC	active	A*02:03 / A*11:01	B*15:02 / B*39:01	Han Chinese
Case12	CBZ	SJS	PBMC	recovery	A*02:01 / A*11:01	B*15:02 / B*40:01	Han Chinese
Case13	CBZ	SJS	PBMC	active	A*02:01 / A*24:02	B*15:02 / B*40:01	Han Chinese
Case14	CBZ	SJS	PBMC	active	A*11:01 / A*11:02	B*15:02 / B*13:01	Han Chinese
Case15	CBZ	SJS	PBMC	recovery	A*02:03 / A*24:02	B*15:02 / B*15:01	Han Chinese
Case16	CBZ	SJS	PBMC	active	A*02:03 / A*02:06	B*15:02 / B*13:01	Han Chinese
Case17	CBZ	SJS	PBMC	active	A*11:01 / A*11:02	B*15:02 / B*15:88	Han Chinese
Case18	CBZ	SJS	PBMC	active	A*02:07 / A*33:03	B*15:02 / B*38:02	Han Chinese
Case19	CBZ	SJS	PBMC	active	A*02:07 / A*11:01	B*15:02 / B*46:01	Han Chinese
Case20	CBZ	SJS	PBMC	recovery	A*24:02 / A*31:01	B*15:02 / B*51:01	Han Chinese
Case21	CBZ	SJS	PBMC	active	A*11:01 / A*24:02	B*15:02 / B*58:01	Han Chinese
Case22	CBZ	SJS	PBMC	recovery	A*11:01 / A*11:02	B*15:02 / B*13:01	Han Chinese
Case23	CBZ	SJS	PBMC	active	A*02:03 / A*33:03	B*15:02 / B*54:01	Han Chinese
Case24	CBZ	SJS	PBMC	recovery	A*11:01 / A*11:01	B*15:02 / B*13:01	Han Chinese
Case25	CBZ	SJS	PBMC	active	A*01:01 / A*66:01	B*07:02 / B*41:02	Europeans
Case26	CBZ	SJS	PBMC	active	A*02:01 / A*11:01	B*15:02 / B*44:02	Europeans
Case27	CBZ	SJS	PBMC	active	A*02:01 / A*23:01	B*41:01 / B*49:01	Europeans
Case28	CBZ	SJS	PBMC	active	A*02:01 / A*11:01	B*15:02 / B*44:02	Europeans
Case29	CBZ	SJS	PBMC	active	A*02:07 / A*34:01	B*15:21 / B*46:01	Europeans
Case30	CBZ	SJS	PBMC	active	A*01:01 / A*03:01	B*08:01 / B*35:03	Europeans
Case31	CBZ	SJS	PBMC	active	A*01:01 / A*01:01	B*08:01 / B*57:01	Europeans
Case32	CBZ	SJS	PBMC	active	A*24:02 / A*26:01	B*35:01 / B*38:01	Europeans
Case33	CBZ	SJS	PBMC	active	A*02:01 / A*03:01	B*07:02 / B*57:01	Europeans
Case34	CBZ	SJS	PBMC	active	A*01:01 / A*31:01	B*57:01 / B*57:01	Europeans
Case35	CBZ	SJS	PBMC	active	A*01:01 / A*30:02	B*08:01 / B*55:01	Europeans
Case36	CBZ	SJS	PBMC	active	A*01:01 / A*31:01	B*13:01 / B*57:01	Europeans
Case37	CBZ	SJS	PBMC	active	A*01:01 / A*66:01	B*08:01 / B*41:02	Europeans
Case38	CBZ	TEN	PBMC	recovery	n.a	B*13:01 / B*15:35	Thai people
Case39	CBZ	SJS	PBMC	recovery	n.a	B*15:02 / B*15:35	Thai people
Case40	CBZ	TEN	PBMC	recovery	n.a	B*15:02 / B*38:02	Thai people
Case41	CBZ	SJS	PBMC	recovery	n.a	B*15:02 / B*39:09	Thai people
Case42	CBZ	TEN	PBMC	recovery	n.a	B*15:02 / B*40:01	Thai people

Male/Female ratio: 0.81/1. Mean age ± s.d. (y): 53.1 ± 15.3. Abbreviations: BC, blister cells; CBZ, carbamazepine; n.a; not available; PBMC, peripheral blood mononuclear cells; SJS, Stevens-Johnson syndrome; TEN, toxic epidermal necrosis.

Supplementary Table 2. Demographics and *HLA-A* and *HLA-B* genotypes of enrolled patients with OXC-, LTG-, PHT-, or ALP-induced SJS/TEN and CBZ-induced DRESS

Patient ID	Culprit drug	Clinical diagnosis	Samples	Disease Stage for experiments	*HLA-A* genotype	*HLA-B* genotype	Populations
Case43	OXC	SJS	BC	active	n.a	B*15:02 / B*44:03	Han Chinese
Case44	OXC	SJS	PBMC	active	A*11:01 / A*11:01	B*15:02 / B*35:01	Han Chinese
Case45	OXC	SJS	PBMC	active	A*11:02 / A*33:03	B*15:02 / B*58:01	Han Chinese
Case46	LTG	SJS	BC	active	A*11:01 / A*31:01	B*15:02 / B*15:27	Han Chinese
Case47	LTG	SJS	BC	active	A*02:06 / A*02:07	B*46:01 / B*48:03	Han Chinese
Case48	LTG	SJS	PBMC	active	A*02:07 / A*24:02	B*39:01 / B*46:01	Han Chinese
Case49	LTG	SJS	PBMC	active	A*33:03 / A*33:03	B*58:01 / B*58:01	Han Chinese
Case50	PHT	TEN	BC	active	A*11:01 / A*11:01	B*15:02 / B*51:01	Han Chinese
Case51	PHT	SJS	BC	active	A*11:01 / A*33:03	B*13:01 / B*58:01	Han Chinese
Case52	PHT	SJS	BC	active	A*11:02 / A*24:02	B*15:02 / B*51:01	Han Chinese
Case53	PHT	SJS	BC	active	A*02:03 / A*11:01	B*38:02 / B*40:01	Han Chinese
Case54	PHT	SJS	BC	active	A*02:03 / A*11:01	B*38:02 / B*58:01	Han Chinese
Case55	PHT	SJS	BC	active	A*02:01 / A*11:01	B*15:02 / B*54:01	Han Chinese
Case56	ALP	TEN	BC	active	n.a	B*58:01 / B*46:01	Han Chinese
Case57	ALP	TEN	BC	active	A*33:03 / A*33:03	B*58:01 / B*58:01	Han Chinese
Case58	ALP	SJS	PBMC	active	n.a	B*58:01 / B*46:01	Han Chinese
Case59	ALP	SJS/TEN	PBMC	active	A*11:01 / A*32:01	B*58:01 / B*38:02	Han Chinese
Case60	ALP	SJS	PBMC	active	n.a	B*58:01 / B*51:01	Han Chinese
Case61	ALP	SJS	PBMC	active	n.a	B*58:01 / B*35:01	Han Chinese
Case62	ALP	SJS	BC	active	n.a	B*58:01 / B*15:02	Han Chinese
Case63	ALP	TEN	BC	active	n.a	B*58:01 / B*35:01	Han Chinese
Case64	ALP	SJS/TEN	BC	active	n.a	B*58:01 / B*13:01	Han Chinese
Case65	ALP	SJS	PBMC	active	n.a	B*58:01 / B*40:01	Han Chinese
Case66	CBZ	DRESS	PBMC	active	A*11:01 / A*31:01	B*13:01 / B*29:01	Han Chinese
Case67	CBZ	DRESS	PBMC	active	A*02:07 / A*31:01	B*46:01 / B*51:01	Han Chinese
Case68	CBZ	DRESS	PBMC	active	A*31:01 / A*33:03	B*15:01 / B*48:01	Han Chinese
Case69	CBZ	DRESS	PBMC	active	A*11:01 / A*11:02	B*27:04 / B*38:01	Han Chinese
Case70	CBZ	DRESS	PBMC	active	A*02:01 / A*02:07	B*46:01 / B*46:01	Han Chinese
Case71	CBZ	DRESS	PBMC	active	A*24:02 / A*31:01	B*15:02 / B*51:84	Han Chinese
Case72	CBZ	DRESS	PBMC	recovery	A*02:01 / A*11:01	B*27:01 / B*35:01	Han Chinese
Case73	CBZ	DRESS	PBMC	recovery	A*11:01 / A*11:01	B*27:04 / B*51:02	Han Chinese

Male/Female ratio: 0.94/1. Mean age ± s.d. (y): 52.4 ± 18.9. Abbreviations: ALP: allopurinol; BC, blister cells; CBZ, carbamazepine; DRESS, drug rash with eosinophilia and systemic symptoms; LTG, lamotrigine; n.a; not available; PBMC, peripheral blood mononuclear cells; OXC, oxcarbazepine; PHT, phenytoin; SJS, Stevens-Johnson syndrome; TEN, toxic epidermal necrosis.

Supplementary Table 3. Demographics and *HLA-A* and *HLA-B* genotypes in CBZ-tolerant controls

Patient ID	Tolerant drug	Clinical diagnosis	*HLA-A* genotype	*HLA-B* genotype	Populations
T1	CBZ	tolerant control	n.a	B*15:02 / B*40:01	Han Chinese
T2	CBZ	tolerant control	n.a	B*15:02 / B*40:01	Han Chinese
T3	CBZ	tolerant control	A*11:01 / A*11:01	B*15:02 / B*13:01	Han Chinese
T4	CBZ	tolerant control	A*11:01 / A*33:03	B*15:02 / B*58:01	Han Chinese
T5	CBZ	tolerant control	A*11:01 / A*24:06	B*15:02 / B*39:01	Han Chinese
T6	CBZ	tolerant control	A*33:03 / A*34:01	B*15:02 / B*56:01	Han Chinese
T7	CBZ	tolerant control	A*11:01 / A*24:02	B*40:01 / B*40:01	Han Chinese
T8	CBZ	tolerant control	A*11:01 / A*24:02	B*15:01 / B*40:01	Han Chinese
T9	CBZ	tolerant control	A*02:01 / A*02:07	B*40:01 / B*46:01	Han Chinese
T10	CBZ	tolerant control	A*24:02 / A*33:03	B*51:01 / B*58:01	Han Chinese
T11	CBZ	tolerant control	A*11:01 / A*30:01	B*13:02 / B*40:02	Han Chinese
T12	CBZ	tolerant control	A*02:01 / A*02:01	B*35:01 / B*40:01	Han Chinese

Male/Female ratio: 1/1. Mean age ± s.d. (y): 45.8 ± 17.2. Abbreviations: CBZ, carbamazepine; F: female; M: male; n.a; not available.

Supplementary Table 4. Demographics and *HLA-A*, *HLA-B* and *HLA-C* genotypes of healthy donor controls.

Donor ID	*HLA-A* genotype	*HLA-B* genotype	*HLA-C* genotype	Populations
HD1	A*02:03 / A*33:03	B*56:01 / B*58:01	C*01:02 / C*03:02	Han Chinese
HD2	A*11:01 / A*34:01	B*15:21 / B*52:01	C*04:03 / C*12:02	Han Chinese
HD3	A*11:01 / A*24:02	B*40:01 / B*40:01	C*07:02 / C*07:02	Han Chinese
HD4	A*02:07 / A*11:01	B*18:02 / B*46:01	C*01:02 / C*07:04	Han Chinese
HD5	A*24:02 / A*33:03	B*40:01 / B*46:01	C*01:02 / C*15:02	Han Chinese
HD6	A*02:01 / A*02:01	B*52:01 / B*55:02	C*03:03 / C*12:02	Han Chinese
HD7	A*11:01 / A*24:02	B*39:01 / B*40:06	C*07:02 / C*08:01	Han Chinese
HD8	A*02:01 / A*02:07	B*39:01 / B*46:01	C*01:02 / C*07:02	Han Chinese
HD9	A*02:06 / A*02:07	B*46:01 / B*56:01	C*01:02 / C*01:02	Han Chinese
HD10	A*11:01 / A*11:01	B*40:01 / B*58:01	C*03:02 / C*07:02	Han Chinese
HD11	A*11:01 / A*11:01	B*13:01 / B*54:01	C*01:02 / C*03:04	Han Chinese
HD12	A*02:03 / A*33:03	B*56:04 / B*58:01	C*01:02 / C*03:02	Han Chinese
HD13	A*11:01 / A*24:02	B*35:01 / B*54:01	C*01:02 / C*03:03	Han Chinese
HD14	A*11:01 / A*24:02	B*38:02 / B*54:01	C*01:02 / C*07:02	Han Chinese
HD15	A*11:01 / A*11:01	B*15:02 / B*40:01	C*07:02 / C*08:01	Han Chinese
HD16	A*02:07 / A*02:06	B*40:01 / B*46:01	C*01:02 / C*03:04	Han Chinese
HD17	A*02:01 / A*11:01	B*40:01 / B*58:01	C*07:02 / C*07:02	Han Chinese
HD18	A*02:03 / A*02:01	B*38:02 / B*52:01	C*07:02 / C*12:02	Han Chinese
HD19	A*02:01 / A*24:02	B*51:02 / B*58:01	C*03:02 / C*15:02	Han Chinese
HD20	A*01:01 / A*02:03	B*38:02 / B*50:01	C*02:02 / C*07:02	Han Chinese
HD21	A*02:01 / A*11:02	B*15:02 / B*15:18	C*08:01 / C*08:01	Han Chinese
HD22	A*24:02 / A*33:03	B*39:05 / B*58:01	C*03:02 / C*07:02	Han Chinese
HD23	A*02:07 / A*11:02	B*27:04 / B*46:01	C*01:02 / C*12:02	Han Chinese
HD24	A*02:03 / A*02:07	B*40:01 / B*46:01	C*01:02 / C*03:04	Han Chinese
HD25	A*02:01 / A*02:07	B*15:01 / B*46:01	C*01:02 / C*04:01	Han Chinese
HD26	A*02:03 / A*02:01	B*40:01 / B*55:02	C*15:02 / C*07:02	Han Chinese
HD27	A*02:03 / A*11:01	B*39:01 / B*40:01	C*07:02 / C*07:02	Han Chinese
HD28	A*24:02 / A*11:02	B*40:01 / B*40:01	C*07:02 / C*07:02	Han Chinese
HD29	A*02:03 / A*02:06	B*51:01 / B*38:02	C*15:02 / C*07:02	Han Chinese
HD30	A*24:02 / A*33:03	B*58:01 / B*39:01	C*07:02 / C*03:02	Han Chinese
HD31	A*02:07 / A*11:01	B*46:01 / B*51:01	C*01:02 / C*14:02	Han Chinese
HD32	A*11:01 / A*11:01	B*15:01 / B*40:02	C*15:02 / C*15:02	Han Chinese
HD33	A*02:07 / A*33:03	B*15:01 / B*58:01	C*03:02 / C*07:02	Han Chinese
HD34	A*11:01 / A*74:02	B*07:02 / B*40:01	C*07:02 / C*07:02	Han Chinese
HD35	A*11:02 / A*02:03	B*15:58 / B*40:01	C*04:03 / C*01:02	Han Chinese
HD36	A*02:07 / A*24:02	B*40:01 / B*46:01	C*01:02 / C*03:04	Han Chinese
HD37	A*11:01 / A*02:03	B*38:02 / B*15:02	C*08:01 / C*07:02	Han Chinese
HD38	A*24:02 / A*02:07	B*46:01 / B*52:01	C*07:02 / C*01:02	Han Chinese
HD39	A*02:07 / A*02:03	B*38:02 / B*46:01	C*07:02 / C*01:02	Han Chinese
HD40	A*11:01 / A*02:07	B*46:01 / B*54:01	C*01:02 / C*01:02	Han Chinese
HD41	A*24:02 / A*11:01	B*39:01 / B*54:01	C*01:02 / C*07:02	Han Chinese
HD42	A*11:02 / A*11:01	B*40:06 / B*40:01	C*07:02 / C*08:01	Han Chinese
HD43	A*11:01 / A*31:01	B*35:01 / B*51:02	C*15:02 / C*03:03	Han Chinese
HD44	A*33:03 / A*11:01	B*15:02 / B*58:01	C*08:01 / C*03:02	Han Chinese

Supplementary Table 5. The "CDR3 ASSLAGELF cluster" and the respective expression frequencies in the blister cells of CBZ-SJS/TEN patients (n=7)

Amino acid sequence	CBZ-BC1	CBZ-BC2	CBZ-BC3	CBZ-BC4	CBZ-BC5	CBZ-BC6	CBZ-BC7
**ASSLAGELF**	3.7284	19.3924	6.6435	42.8493	17.4130	0.1000	3.9950
**ASSLSGELF**	0.0544	0.9964	0.0074	1.9218	0.0354	0.6600	0.0480
**ASSFAGELF**	0.0019	0.2275	0.0061	0.0179	0.0043	1.9724	0.7968
**ASSLGGELF**	0.0021	0.0877	0.0000	0.2449	0.0027	0.5036	0.0142
**ASSSAGELF**	0.0048	0.5466	0.0344	0.0509	0.0348	0.0016	0.0013
**ASSPAGELF**	0.0414	0.5866	0.0000	0.0000	0.0000	0.0000	0.0128
**ASSLAGGLF**	0.0127	0.1035	0.0307	0.1444	0.0855	0.0003	0.0098
**ASSLAGELL**	0.0180	0.0786	0.0098	0.1637	0.0828	0.0003	0.0076
**AGSLAGELF**	0.0153	0.0573	0.0381	0.1032	0.0571	0.0002	0.0095
**ASGLAGELF**	0.0074	0.0937	0.0221	0.0853	0.0526	0.0003	0.0036
**ASSLAGEPF**	0.0075	0.0572	0.0184	0.1032	0.0517	0.0002	0.0078
**Total**	3.8939	22.2276	6.8104	45.6845	17.8198	3.2391	4.9064

The "CDR3 ASSLAGELF cluster" includes similar TCRβ clonotypes with one amino acid residue difference (e.g., "ASSLSGELF", "ASSFAGELF", etc.) identified in the blister cells of patients with CBZ-SJS/TEN. Though the public CDR3 "ASSLAGELF" was predominately detected, other similar clonotypes were also present in the samples.

Supplementary Table 6. Primers/probes used for Taqman real-time PCR

*For detecting the specific TCRβ CDR3 "ASSLAGELF" clonotype:	
forward primer:	5'-TTCTCAGCTAAGATGCCTAATGCA-3'
reverse primer:	5'-AAACAGCTCCCCGGCTAAA-3'
probe:	5'-TGAAGATCCAGCCCTC-3'
*For detecting the specific TCRα CDR3 "VFDNTDKLI" clonotype:	
forward primer:	5'-CTCAGTGATTCAGCCACCTACCT-3'
reverse primer:	5'-TGGTCCCAGTCCCAAAGATG-3'
probe:	5'-TCGATAACACCGACAAGC-3'

Supplementary Table 7. Sequence of cDNA encoding for the soluble single-chain αβTCR (scTCR).

**cDNA sequence of scTCR:**
atgatatccttgagagttttactggtgatcctgtggcttcagttaagctgggtttggagccaacggaaggaggtggagcagg atcctggacccttcaatgttccagagggagccactgtcgctttcaactgtacttacagcaacagtgcttctcagtctttcttctg gtacagacaggattgcaggaaagaacctaagttgctgatgtccgtatactccagtggtaatgaagatggaaggtttacagca cagctcaatagagccagccagtatatttccctgctcatcagagactccaagctcagtgattcagccacctacctctgtgtgttc gataacaccgacaagctcatctttgggactgggaccagattacaagtctttccaaagctttcagggagtgcatccgccccaa aacttgaagaaggtgaattttcagaagcacgcgtagatgctggagttatccagtcaccccggcacgaggtgacagagatg ggacaagaagtgactctgagatgtaaaccaatttcaggacatgactaccctttctggtacagacagaccatgatgcgggga ctggagttgctcatttactttaacaacaacgttccgatagatgattcagggatgcccgaggatcgattctcagctaagatgcct aatgcatcattctccactctgaagatccagccctcagaacccagggactcagctgtgtacttctgtgccagcagtttagcagg ggagctgttttttggagaaggctctaggctgaccgtactggctagcaaatcttctgacaaaactcacacatgcccaccgtgc ccagcacctgaactcctggggggaccgtcagtcttcctcttccccccaaaacccaaggacaccctcatgatctcccggacc cctgaggtcacatgcgtggtggtggacgtgagccacgaagaccctgaggtcaagttcaactggtacgtggacggcgtgg aggtgcataatgccaagacaaagccgcgggaggagcagtacaacagcacgtaccgtgtggtcagcgtcctcaccgtcct gcaccaggactggctgaatggcaaggagtacaagtgcaaggtctccaacaaagccctcccagcccccatcgagaaaac catctccaaagccaaagggcagccccgagaaccacaggtgtacaccctgcccccatcccgggaggagatgaccaagaa ccaggtcagcctgacctgcctggtcaaaggcttctatcccagcgacatcgccgtggagtgggagagcaatgggcagccg gagaacaactacaagaccacgcctcccgtgctggactccgacggctccttcttcctctatagcaagctcaccgtggacaag agcaggtggcagcaggggaacgtcttctcatgctccgtgatgcatgaggctctgcacaaccactacacgcagaagagcct ctccctgtctccgggtaaatga

The scTCR construct composed of TCRα CDR3 “VFDNTDKLI” and TCRβ CDR3 “ASSLAGELF” was cloned from the cDNA of blister cells of a patient with CBZ-induced SJS/TEN (case 4). The TCRα and TCRβ fragments were connected by a linker, and the human antibody Fc region (underline) was introduced behind the TCRα-linker-TCRβ fragment. The single chain TCRα-linker-TCRβ-Fc insert was cloned into a pcDNA vector (pcDNA/scTCR-Fc). The linker sequence: aagctttcagggagtgcatccgccccaaaacttgaagaaggtgaattttcagaagcacgcgta.

Supplementary Table 8. The cDNA sequences of TCRα and TCRβ for generating the TCR transfectants

TCRα:

atgatatccttgagagttttactggtgatcctgtggcttcagttaagctgggtttggagccaacggaaggaggtggagcagg
atcctggacccttcaatgttccagagggagccactgtcgctttcaactgtacttacagcaacagtgcttctcagtctttcttctg
gtacagacaggattgcaggaaagaacctaagttgctgatgtccgtatactccagtggtaatgaagatggaaggtttacagca
cagctcaatagagccagccagtatatttccctgctcatcagagactccaagctcagtgattcagccacctacctctgtgtgttc
gataacaccgacaagctcatctttgggactgggaccagattacaagtctttccaaatatccagaatccggaacctgctgtgta
ccagttaaaagatcctcggtctcaggacagcaccctctgcctgttcaccgactttgactcccaaatcaatgtgccgaaaacc
atggaatctggaacgttcatcactgacaaaactgtgctggacatgaaagctatggattccaagagcaatggggccattgcct
ggagcaaccagacaagcttcacctgccaagatatcttcaaagagaccaacgccacctaccccagttcagacgttccctgtg
atgccacgttgaccgagaaaagctttgaaacagatatgaacctaaactttcaaaacctgtcagttatgggactccgaatcctc
ctgctgaaagtagcgggatttaacctgctcatgacgctgaggctgtggtccagttgag

TCRβ:

atggactcctggaccctctgctgtgtgtccctttgcatcctggtagcaaagcacacagatgctggagttatccagtcaccccg
gcacgaggtgacagagatgggacaagaagtgactctgagatgtaaaccaatttcaggacatgactaccctttctggtacag
acagaccatgatgcggggactggagttgctcatttactttaacaacaacgttccgatagatgattcagggatgcccgaggat
cgattctcagctaagatgcctaatgcatcattctccactctgaagatccagccctcagaacccagggactcagctgtgtactt
ctgtgccagcagtttagcaggggagctgtttttggagaaggctctaggctgaccgtactggaggatctgagaaatgtgact
ccacccaaggtctccttgtttgagccatcaaaagcagagattgcaaacaaacaaaaggctaccctcgtgtgcttggccagg
ggcttcttccctgaccacgtggagctgagctggtgggtgaatggcaaggaggtccacagtggggtcagcacggaccctc
aggcctacaaggagagcaattatagctactgcctgagcagccgcctgagggtctctgctaccttctggcacaatcctcgaa
accacttccgctgccaagtgcagttccatgggctttcagaggaggacaagtggccagagggctcacccaaacctgtcaca
cagaacatcagtgcagaggcctggggccgagcagactgtggaatcacttcagcatcctatcatcaggggggttctgtctgca
accatcctctatgagatcctactggggaaggccaccctatatgctgtgctggtcagtggcctggtgctgatggccatggtca
agaaaaaaaaattcctg

The cDNA sequences containing the TCRα CDR3 “VFDNTDKLI” and the TCRβ CDR3 “ASSLAGELF” were obtained and cloned from the samples of blister cells of a CBZ-SJS/TEN patient (case 4). The unique TCR α or β CDR3 and VDJ regions were linked to the mouse TCR α or β constant domain (underline), respectively. The hybridized TCR α or β fragments was cloned into MSCV-based retroviral vector for generating 5KC-TCR transfectants.
