# Supplementary material for: Identification of drug-specific public TCR driving severe cutaneous adverse reactions
Source: Nat Commun. 2019 Aug 8;10:3569. doi: 10.1038/s41467-019-11396-2 (PMC6687717; doi:10.1038/s41467-019-11396-2)
Supplement: Supplementary file 3 — Reporting Summary [file 41467_2019_11396_MOESM3_ESM.pdf]

nature research

Corresponding author(s): Shuen-Iu Hung, Wen-Hung Chung

Last updated by author(s): Jun 24, 2019

Reporting Summary

Nature Research wishes to improve the reproducibility of the work that we publish. This form provides structure for consistency and transparency in reporting. For further information on Nature Research policies, see Authors & Referees and the Editorial Policy Checklist.

Statistics

For all statistical analyses, confirm that the following items are present in the figure legend, table legend, main text, or Methods section.

n/a	Confirmed	
☐	☒	The exact sample size (n) for each experimental group/condition, given as a discrete number and unit of measurement
☐	☒	A statement on whether measurements were taken from distinct samples or whether the same sample was measured repeatedly
☐	☒	The statistical test(s) used AND whether they are one- or two-sided<br>*Only common tests should be described solely by name; describe more complex techniques in the Methods section.*
☒	☐	A description of all covariates tested
☐	☒	A description of any assumptions or corrections, such as tests of normality and adjustment for multiple comparisons
☐	☒	A full description of the statistical parameters including central tendency (e.g. means) or other basic estimates (e.g. regression coefficient) AND variation (e.g. standard deviation) or associated estimates of uncertainty (e.g. confidence intervals)
☒	☐	For null hypothesis testing, the test statistic (e.g. F, t, r) with confidence intervals, effect sizes, degrees of freedom and P value noted<br>*Give P values as exact values whenever suitable.*
☒	☐	For Bayesian analysis, information on the choice of priors and Markov chain Monte Carlo settings
☒	☐	For hierarchical and complex designs, identification of the appropriate level for tests and full reporting of outcomes
☒	☐	Estimates of effect sizes (e.g. Cohen's d, Pearson's r), indicating how they were calculated

Our web collection on statistics for biologists contains articles on many of the points above.

Software and code

Policy information about availability of computer code

Data collection	PCR amplification and next generation sequencing (NGS) for the TCR repertoire: Illumina Miseq<br>flow cytometry: Cytomics FC500 flow cytometer, CXP software (Beckman Coulter)<br>Surface plasmon resonance analysis: Biacore T200 surface plasmon resonance (SPR) biosensor<br>IL-2 production: enzyme-linked immunospot (ELISPOT) assay (Mabtech) and an ImmunoSpot reader (CTL Cellular Technology)
Data analysis	The TRBV, TRBJ, and CDR3 clonotypes were defined according to the ImMunoGeneTics information (IMGT) database (www.imgt.org).<br>Significant differences between the groups were analyzed using an unpaired, two-tailed Student's t test.<br>Graphs were produced using Graphpad Prism (version 7.02).<br>The heat maps were generated using the built-in R heatmap() function in stats package.<br>The Circos plots were generated by VDJtools software (MiLaboratory).<br>The treemaps were generated by the treemap() function version 1.2.0.1, MATLAB Central File Exchange.

For manuscripts utilizing custom algorithms or software that are central to the research but not yet described in published literature, software must be made available to editors/reviewers. We strongly encourage code deposition in a community repository (e.g. GitHub). See the Nature Research guidelines for submitting code & software for further information.

Data

Policy information about availability of data

All manuscripts must include a data availability statement. This statement should provide the following information, where applicable:

- Accession codes, unique identifiers, or web links for publicly available datasets
- A list of figures that have associated raw data
- A description of any restrictions on data availability

The source data underlying Figs 1a, 1b, 2a, 2c, 2f, 3a, 3b, 3c, 3d, 3e, 3f, 4b, 4c, 5, 6a, 6c, 6d, 7f-j, 7k-m, and Supplementary Figures 1, 2, 8, 10, 12 are provided as the Source Data file. The sequence data that support the findings of this study have been deposited in the NCBI sequence read archive (SRA) database with links to

BioProject accession number “PRJNA550004”.

Field-specific reporting

Please select the one below that is the best fit for your research. If you are not sure, read the appropriate sections before making your selection.

☒ Life sciences ☐ Behavioural & social sciences ☐ Ecological, evolutionary & environmental sciences

For a reference copy of the document with all sections, see nature.com/documents/nr-reporting-summary-flat.pdf

Life sciences study design

All studies must disclose on these points even when the disclosure is negative.

Sample size	We enrolled a total of 129 subjects, including 73 patients with SCAR (65 with SJS/TEN and 8 with DRESS), 12 tolerant controls, and 44 healthy donors of Han Chinese
Data exclusions	No data were excluded.
Replication	The identified NGS TCR result was further confirmed by TaqMan qPCR assay as well as by other functional assays. The developed software produces the same NGS results when run with the same starting conditions/parameters.
Randomization	Does not apply. This work does not describe an experimental study that requires allocation into groups.
Blinding	Does not apply. This work does not describe an experimental study that requires allocation into groups

Behavioural & social sciences study design

All studies must disclose on these points even when the disclosure is negative.

Study description	*Briefly describe the study type including whether data are quantitative, qualitative, or mixed-methods (e.g. qualitative cross-sectional, quantitative experimental, mixed-methods case study).*
Research sample	*State the research sample (e.g. Harvard university undergraduates, villagers in rural India) and provide relevant demographic information (e.g. age, sex) and indicate whether the sample is representative. Provide a rationale for the study sample chosen. For studies involving existing datasets, please describe the dataset and source.*
Sampling strategy	*Describe the sampling procedure (e.g. random, snowball, stratified, convenience). Describe the statistical methods that were used to predetermine sample size OR if no sample-size calculation was performed, describe how sample sizes were chosen and provide a rationale for why these sample sizes are sufficient. For qualitative data, please indicate whether data saturation was considered, and what criteria were used to decide that no further sampling was needed.*
Data collection	*Provide details about the data collection procedure, including the instruments or devices used to record the data (e.g. pen and paper, computer, eye tracker, video or audio equipment) whether anyone was present besides the participant(s) and the researcher, and whether the researcher was blind to experimental condition and/or the study hypothesis during data collection.*
Timing	*Indicate the start and stop dates of data collection. If there is a gap between collection periods, state the dates for each sample cohort.*
Data exclusions	*If no data were excluded from the analyses, state so OR if data were excluded, provide the exact number of exclusions and the rationale behind them, indicating whether exclusion criteria were pre-established.*
Non-participation	*State how many participants dropped out/declined participation and the reason(s) given OR provide response rate OR state that no participants dropped out/declined participation.*
Randomization	*If participants were not allocated into experimental groups, state so OR describe how participants were allocated to groups, and if allocation was not random, describe how covariates were controlled.*

Ecological, evolutionary & environmental sciences study design

All studies must disclose on these points even when the disclosure is negative.

Study description	*Briefly describe the study. For quantitative data include treatment factors and interactions, design structure (e.g. factorial, nested, hierarchical), nature and number of experimental units and replicates.*
Research sample	*Describe the research sample (e.g. a group of tagged Passer domesticus, all Stenocereus thurberi within Organ Pipe Cactus National Monument), and provide a rationale for the sample choice. When relevant, describe the organism taxa, source, sex, age range and*

any manipulations. State what population the sample is meant to represent when applicable. For studies involving existing datasets, describe the data and its source.

Sampling strategy	*Note the sampling procedure. Describe the statistical methods that were used to predetermine sample size OR if no sample-size calculation was performed, describe how sample sizes were chosen and provide a rationale for why these sample sizes are sufficient.*
Data collection	*Describe the data collection procedure, including who recorded the data and how.*
Timing and spatial scale	*Indicate the start and stop dates of data collection, noting the frequency and periodicity of sampling and providing a rationale for these choices. If there is a gap between collection periods, state the dates for each sample cohort. Specify the spatial scale from which the data are taken*
Data exclusions	*If no data were excluded from the analyses, state so OR if data were excluded, describe the exclusions and the rationale behind them, indicating whether exclusion criteria were pre-established.*
Reproducibility	*Describe the measures taken to verify the reproducibility of experimental findings. For each experiment, note whether any attempts to repeat the experiment failed OR state that all attempts to repeat the experiment were successful.*
Randomization	*Describe how samples/organisms/participants were allocated into groups. If allocation was not random, describe how covariates were controlled. If this is not relevant to your study, explain why.*
Blinding	*Describe the extent of blinding used during data acquisition and analysis. If blinding was not possible, describe why OR explain why blinding was not relevant to your study.*

Did the study involve field work? ☐ Yes ☒ No

Reporting for specific materials, systems and methods

We require information from authors about some types of materials, experimental systems and methods used in many studies. Here, indicate whether each material, system or method listed is relevant to your study. If you are not sure if a list item applies to your research, read the appropriate section before selecting a response.

Materials & experimental systems

n/a	Involved in the study	
☐	☒	Antibodies
☐	☒	Eukaryotic cell lines
☒	☐	Palaeontology
☐	☒	Animals and other organisms
☐	☒	Human research participants
☐	☒	Clinical data

Methods

n/a	Involved in the study	
☒	☐	ChIP-seq
☐	☒	Flow cytometry
☒	☐	MRI-based neuroimaging

Antibodies

Antibodies used	(Beckman Coulter) anti-human-CD4-ECD (cat: 6604727) (clone: SFCI12T4D11)<br>(Beckman Coulter) anti-human-CD8-FITC (cat: 6602385) (clone: SFCI21Thy2D3)<br>(Beckman Coulter) anti-human-CD56-PC5.5 (cat: B49189) (clone: N901)<br>(Beckman Coulter) anti-human-TRBV12-3/TRBV12-4-PE (cat: IM2289) (clone: 56C5.2)<br>(BioLegend) anti-human-CD45-APC (cat:304012) (clone: HI30)<br>(BioLegend) anti-human-CD45RA-FITC (cat: 304114) (clone: HI100)<br>(BioLegend) anti-human-CD197-PC7 (cat: 353226) (clone: G043H7)<br>(eBioscience) anti-mouse-CD4-APC (cat: 61-0042-82) (clone: RM4-5)<br>(eBioscience) anti-mouse-CD8-FITC (cat: 25-0081-82) (clone: 53-6.7)<br>(eBioscience) anti-mouse- IFNg-PC7 (cat: 61-7311-82) (clone: XMG1.2)
Validation	All antibodies were commercially available, and specificity had been described by the manufacturer.

Eukaryotic cell lines

Policy information about cell lines

Cell line source(s)	ATCC
Authentication	CVCL_6642, CVCL_3714
Mycoplasma contamination	No mycoplasma contamination
Commonly misidentified lines<br>(See ICLAC register)	HEK293F, HMy2.C1R cells, 5KC cells

Animals and other organisms

Policy information about studies involving animals; ARRIVE guidelines recommended for reporting animal research

Laboratory animals	mouse
Wild animals	We generated the mono-chain homozygous HLA-B*15:02 transgenic mice in the C57BL/6 genetic background with triple-knockout of the mouse MHC class I genes H-2b and H-2d, and B2-microglobulin genes. The stable expression of the HLA-B*15:02 protein in the transgenic mice was confirmed in this study.
Field-collected samples	Skin, blood
Ethics oversight	The Experimental Animal Ethics Committee of the institute (National Yang-Ming University) approved the animal protocols of this study (IACUC Approval No.: 1031232 and 1041238)

Note that full information on the approval of the study protocol must also be provided in the manuscript.

Human research participants

Policy information about studies involving human research participants

Population characteristics	We enrolled a total of 129 subjects, including 73 patients with SCAR (65 with SJS/TEN and 8 with DRESS), 12 tolerant controls, and 44 healthy donors of Han Chinese.
Recruitment	Among 73 patients with SJS/TEN or DRESS, there were 55 Chinese enrolled from the Chang Gung Memorial Hospital Health System and Taiwan severe cutaneous adverse reactions (T-SCAR) consortium (including National Taiwan University Hospital, Taichung Veterans General Hospital, National Cheng Kung University Hospital, and Kaohsiung Medical University, and Chung-Ho Memorial Hospital) in Taiwan, 5 from Thailand, and 13 recruited by the RegiSCAR group from Europe, during 2011 and 2018.
Ethics oversight	Approval for the study was obtained from the institutional review board (103-2562C, 104-2664A3, 201601761BO, YM106026F-1, IRB00001189, R.1235-9), and informed consent was obtained from each participant.

Note that full information on the approval of the study protocol must also be provided in the manuscript.

Clinical data

Policy information about clinical studies

All manuscripts should comply with the ICMJE guidelines for publication of clinical research and a completed CONSORT checklist must be included with all submissions.

Clinical trial registration	*Provide the trial registration number from ClinicalTrials.gov or an equivalent agency.*
Study protocol	*Note where the full trial protocol can be accessed OR if not available, explain why.*
Data collection	*Describe the settings and locales of data collection, noting the time periods of recruitment and data collection.*
Outcomes	*Describe how you pre-defined primary and secondary outcome measures and how you assessed these measures.*

Flow Cytometry

Plots

Confirm that:

- [x] The axis labels state the marker and fluorochrome used (e.g. CD4-FITC).
- [x] The axis scales are clearly visible. Include numbers along axes only for bottom left plot of group (a 'group' is an analysis of identical markers).
- [x] All plots are contour plots with outliers or pseudocolor plots.
- [x] A numerical value for number of cells or percentage (with statistics) is provided.

Methodology

Sample preparation	The PBMC or blister cells were repsupend in PBS, and then was blocked by 0.5%BSA/PBS for 30 mins. The blocked cells were stained with different fluorescence-conjugated mAbs for 30 mins on ice. After two times of wash, the cells are ready for flow cytometry analysis.
Instrument	Beckman Coulter FC500 flow cytometer
Software	Beckman Coulter CXP software

Cell population abundance	The cellular abundance of this study was CD3+ T cells.
Gating strategy	The cells were gated on FSC-A/FSC-H to select the single cells, and then were gated on FSC-A/SSC-A to select the lymphocytes as the starting cell population.

☒ Tick this box to confirm that a figure exemplifying the gating strategy is provided in the Supplementary Information.
